# Endoscopic excision for posterior ankle impingement secondary to chronic fragmented os trigonum fracture: a case report

**DOI:** 10.1093/jscr/rjag577

**Published:** 2026-07-13

**Authors:** Thang Ngoc Pham, Anh The Nguyen, Anh Tuan Bui

**Affiliations:** Orthopaedic Trauma Center, 103 Military Hospital, Vietnam Military Medical University, Ha Dong, Hanoi 100000, Vietnam; Department of Surgery, Thai Nguyen University of Medicine and Pharmacy, Phan Dinh Phung, Thai Nguyen 250000, Vietnam; Orthopaedic Trauma Center, 103 Military Hospital, Vietnam Military Medical University, Ha Dong, Hanoi 100000, Vietnam

**Keywords:** os trigonum fracture, posterior ankle impingement syndrome, posterior ankle endoscopy, flexor hallucis longus

## Abstract

Chronic fragmented os trigonum fracture is an uncommon but clinically important cause of posterior ankle impingement that may be overlooked in active patients with persistent plantar-flexion-related posterior ankle pain. We report the case of a 31-year-old female physical education teacher with progressive right posterior ankle pain for over 1 year despite 3 months of conservative treatment. Radiographs and magnetic resonance imaging demonstrated a fragmented os trigonum with bone marrow edema and reactive inflammation. Posterior ankle endoscopy confirmed three osseous fragments, which were excised sequentially. Pain resolved within 3 weeks, light running resumed at 8 weeks, and full return to preinjury sports was achieved at 3 months. At 5-year follow-up, the patient remained asymptomatic with no recurrence and complete functional recovery. This case highlights the importance of considering this diagnosis in active patients with persistent posterior ankle pain and supports posterior ankle endoscopy as an effective treatment with durable results.

## Introduction

Posterior ankle impingement syndrome is a recognized cause of chronic posterior ankle pain, particularly in athletes and physically active individuals performing repetitive forced plantar flexion. It results from compression of osseous or soft-tissue structures between the posterior tibia and posterior talus during terminal plantar flexion. Common osseous causes include a symptomatic os trigonum and a prominent posterior talar process, whereas soft-tissue causes include flexor hallucis longus (FHL) pathology, scar tissue, and posterior capsuloligamentous impingement [1–4].

The os trigonum is an accessory ossicle posterior to the talus and is usually asymptomatic [3, 4]. In susceptible individuals, however, it may become symptomatic and cause posterior ankle pain, particularly during activities requiring repetitive plantar flexion [1, 4, 5]. True fracture or chronic fragmentation of a pre-existing os trigonum is uncommon and may be overlooked, especially in active patients without a clear history of acute trauma [6–8]. Because the clinical presentation overlaps with more common causes of posterior ankle pain, including Achilles-related disorders, FHL pathology, and posterior talar process injury, diagnosis may be delayed. Recognition of this entity is important, as persistent mechanical impingement may lead to chronic pain and prolonged limitation of sports participation. We report a rare case of chronic posterior ankle impingement secondary to fragmented os trigonum fracture in a recreational athlete, successfully treated by posterior ankle endoscopy, with an excellent 5-year outcome.

## Case report

A 31-year-old female physical education teacher and recreational athlete presented with progressive right posterior ankle pain of more than 1 year’s duration. The pain had an insidious onset without a definite history of acute trauma. Symptoms were aggravated by sprinting, uphill running, jumping, and activities requiring terminal plantar flexion. Rest provided partial relief, but symptoms consistently recurred upon return to physical activity, ultimately preventing participation in sports at her previous level.

Before surgical consideration, the patient underwent 3 months of conservative treatment, including activity modification, temporary cessation of sports, non-steroidal anti-inflammatory drugs, ankle immobilization, and physiotherapy. Despite compliance with this programme, symptoms improved only marginally, and posterior ankle pain persisted during exertion. Physical examination revealed focal tenderness in the posterior ankle, immediately anterior to the Achilles tendon insertion. Forced passive plantar flexion reproduced the pain. Anterior drawer and talar tilt tests were negative, and no neurovascular abnormality was identified. The preoperative American Orthopaedic Foot and Ankle Society (AOFAS) ankle-hindfoot score was 75/100, and the visual analogue scale (VAS) pain score was 6/10.

**Figure 1:**
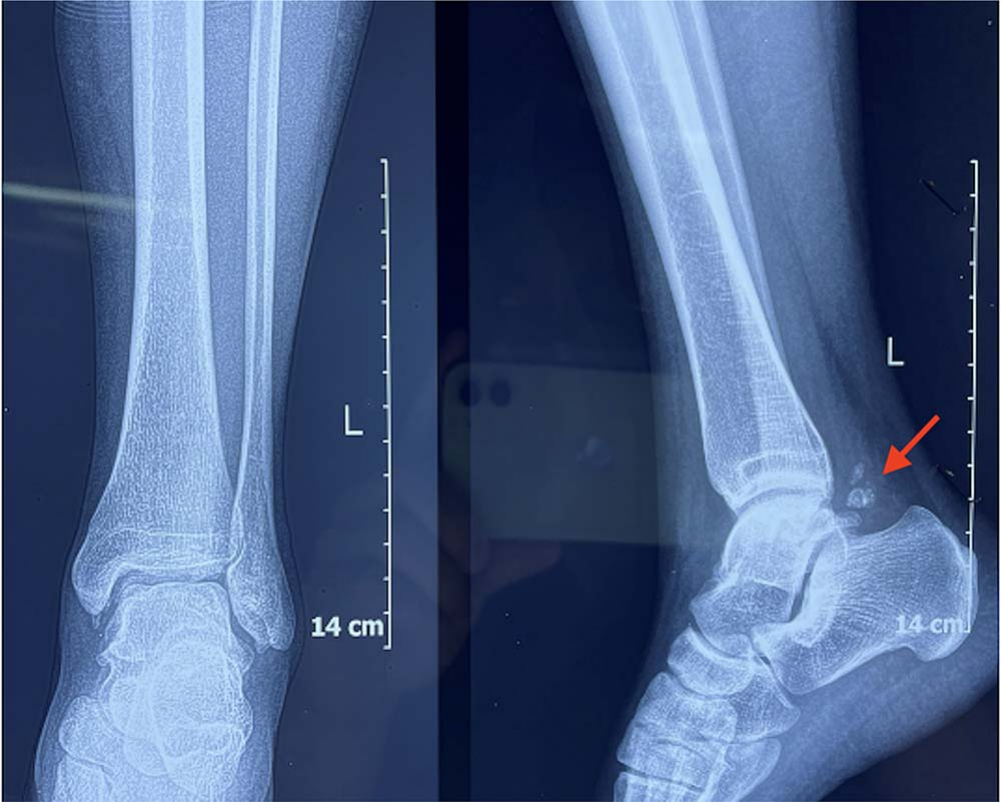
Lateral weight-bearing radiograph of the right ankle demonstrating a fragmented os trigonum posterior to the talus (arrow).

**Figure 2:**
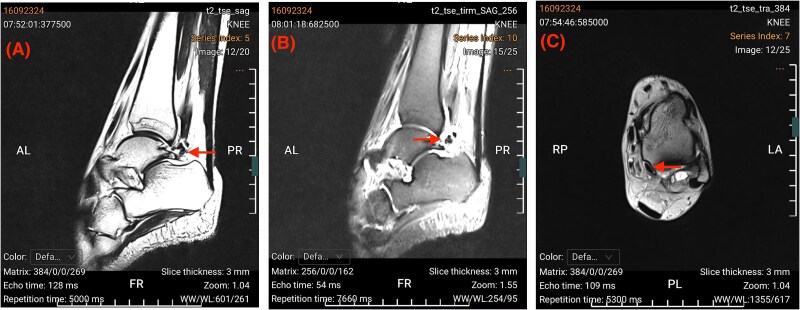
Preoperative MRI of the right ankle. (A) Sagittal T2-weighted image demonstrating a fragmented os trigonum posterior to the talus (arrow). (B) Sagittal fat-suppressed Turbo Inversion Recovery Magnitude image showing bone marrow oedema within the os trigonum fragments and surrounding soft-tissue inflammatory change (arrow). (C) Axial T2-weighted image demonstrating fluid surrounding the FHL tendon sheath (arrow).

Lateral weight-bearing ankle radiographs demonstrated several well-corticated ossified fragments posterior to the talus, consistent with a fragmented os trigonum (Fig. 1). Magnetic resonance imaging (MRI) demonstrated a main os trigonum fragment with two adjacent detached fragments, associated bone marrow oedema, and reactive soft-tissue inflammatory change, consistent with a symptomatic lesion (Fig. 2). The FHL tendon was intact, with no evidence of partial tear. Given the persistent functional limitation, characteristic clinical and imaging findings, and failure of conservative treatment, chronic posterior ankle impingement secondary to fragmented os trigonum fracture was diagnosed, and endoscopic excision was indicated.

### Surgical technique

**Figure 3:**
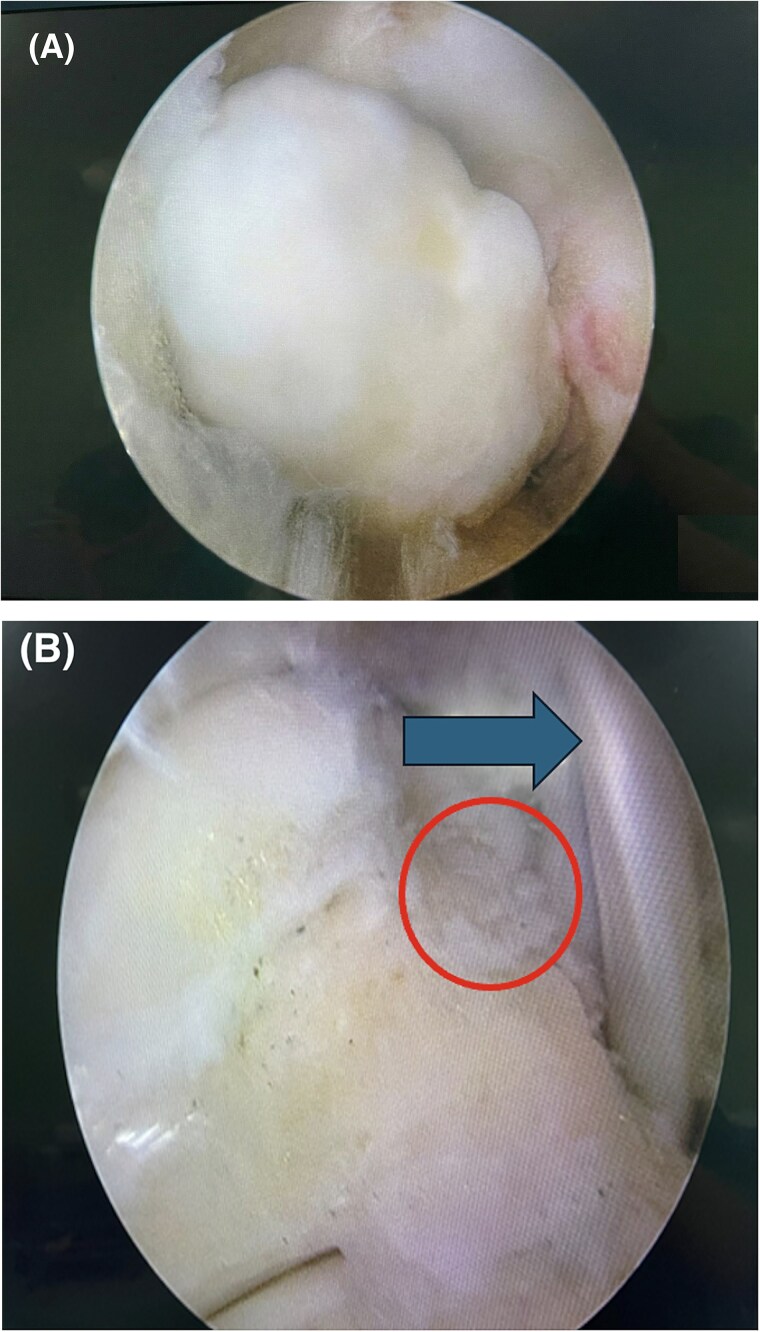
Intraoperative endoscopic views of the posterior right ankle. (A) Endoscopic view demonstrating one of the osseous fragments before excision. (B) Endoscopic view after complete excision, showing restoration of a smooth posterior talar contour (circle). The arrow indicates the intact FHL tendon, which served as the medial anatomic landmark.

The procedure was performed under spinal anaesthesia with the patient in the prone position. Standard posterolateral and posteromedial portals were established just proximal to the intermalleolar line, immediately adjacent to the Achilles tendon. Using a 4.0-mm, 30-degree arthroscope, the posterior ankle compartment was accessed, and the soft tissue surrounding the os trigonum was debrided. Intraoperatively, the os trigonum was confirmed to be fragmented into three distinct osseous fragments, which were excised sequentially under direct endoscopic visualization (Fig. 3A). The FHL tendon was identified and served as the key medial anatomic landmark throughout the procedure. All instrumentation was kept lateral to the tendon to protect the neurovascular bundle. Although preoperative MRI suggested mild FHL sheath inflammation, the tendon glided smoothly after fragment removal, and no tendon sheath release was required (Fig. 3B). Intraoperative fluoroscopy and dynamic assessment during terminal plantar flexion confirmed complete removal of all fragments and resolution of posterior osseous impingement. No additional pathology requiring intervention was identified.

### Postoperative rehabilitation and outcome

Recovery was uneventful. Near-normal walking was achieved by 2 weeks, and pain resolved completely within 3 weeks, with the VAS pain score decreasing to 0/10. Light running was initiated at 8 weeks, and full return to preinjury sporting activity was achieved at 3 months. No postoperative complications, including wound problems, nerve injury, or prolonged stiffness, were observed. At 5-year follow-up, the patient remained asymptomatic, with an AOFAS score of 100/100 and a VAS pain score of 0/10. She reported no residual functional limitation and no recurrence of symptoms. The postoperative recovery timeline and clinical milestones are summarized in Table 1.

**Table 1:** Postoperative recovery timeline and clinical milestones

Time point	Rehabilitation milestone/functional activity	Clinical status
2 weeks	Near-normal walking resumed	Marked pain reduction; no early postoperative complications
2–3 weeks	Progression of range-of-motion exercises	Complete pain relief (VAS 0/10)
8 weeks	Light running initiated	No recurrence of pain during running
3 months	Full return to preinjury sporting activity	Complete functional recovery
5 years	Continued recreational sports participation	Asymptomatic; AOFAS 100/100; VAS 0/10; no recurrence

## Discussion

Although posterior ankle impingement is well recognized, chronic fragmented os trigonum fracture is a rare cause that may be easily overlooked in active patients without a definite traumatic event. Persistent posterior ankle pain aggravated by terminal plantar flexion should prompt consideration of os trigonum-related impingement, particularly when symptoms fail to respond to conservative treatment [1, 2, 5].

Diagnosis depends on careful correlation between symptoms, physical examination, and imaging findings. In the present case, radiographs demonstrated multiple ossified fragments posterior to the talus, while MRI showed bone marrow oedema and reactive soft-tissue inflammatory change, supporting a symptomatic lesion rather than an incidental accessory ossicle. Differentiation from posterior talar process fracture and multipartite os trigonum is important in this setting [6–8].

Open excision has traditionally been used for symptomatic os trigonum and other posterior impingement lesions. However, posterior ankle endoscopy offers several recognized advantages, including smaller incisions, reduced soft-tissue trauma, direct visualization of the posterior ankle compartment, assessment of associated pathology, and faster rehabilitation [5, 9, 10]. Guo *et al.*, in a comparative study of 41 cases, reported favourable outcomes with endoscopic excision and highlighted the minimally invasive advantages of the endoscopic approach over open surgery [11]. In the present case, the endoscopic approach was particularly advantageous because it allowed direct confirmation and sequential excision of all three osseous fragments, dynamic assessment of impingement, and inspection of the FHL tendon through a minimally invasive approach. Identification of the FHL tendon was especially important, as it served as the key medial anatomic landmark throughout the procedure; instrumentation medial to the tendon would have increased the risk of injury to the posterior tibial neurovascular bundle.

Previous studies have reported favourable outcomes and return to sport after endoscopic treatment of posterior ankle impingement lesions [5, 10, 12]. In the present case, the patient experienced rapid recovery, early return to activity, and durable symptom resolution at 5 years. This recurrence-free long-term outcome is a notable strength of the report, as robust long-term data for this specific presentation remain limited.

This report is limited by its single-patient design and the absence of computed tomography for more detailed fracture characterization. Nevertheless, it remains clinically instructive because of the rarity of the lesion, the diagnostic challenge in an active patient without clear trauma, the consistent clinical and imaging correlation, and the excellent long-term functional outcome following endoscopic treatment.
